# Protective Potential of *Saussurea costus* (Falc.) Lipsch. Roots against Cyclophosphamide-Induced Pulmonary Injury in Rats and Its In Vitro Antiviral Effect

**DOI:** 10.3390/ph16020318

**Published:** 2023-02-18

**Authors:** Nashwah G. M. Attallah, Amal Kabbash, Walaa A. Negm, Engy Elekhnawy, Reem Binsuwaidan, Omnia Momtaz Al-Fakhrany, Moataz A. Shaldam, Ehssan Moglad, Marwa Tarek, Nehal Samir, Heba M. Fawzy

**Affiliations:** 1 The Egyptian Drug Authority (EDA), Previously NODCAR, Giza 8655, Egypt; 2 Department of Pharmacognosy, Faculty of Pharmacy, Tanta University, Tanta 31527, Egypt; 3 Department of Pharmaceutical Microbiology, Faculty of Pharmacy, Tanta University, Tanta 31527, Egypt; 4 Department of Pharmaceutical Science, College of Pharmacy, Princess Nourah bint Abdulrahman University, Riyadh 11671, Saudi Arabia; 5 Department of Pharmaceutical Chemistry, Faculty of Pharmacy, Kafrelsheikh University, Kafr El-Sheikh 33516, Egypt; 6 Department of Pharmaceutics, College of Pharmacy, Prince Sattam bin Abdulaziz University, Alkharj 11942, Saudi Arabia; 7 Medical Biochemistry and Molecular Biology Department, Faculty of Medicine, Ain Shams University, Cairo 11865, Egypt; 8 Histology and Cell Biology Department, Faculty of Medicine, Ain Shams University, Cairo 11865, Egypt

**Keywords:** coronaviruses, influenza virus, oxidative stress, histology, microRNAs, molecular docking

## Abstract

Diseases and infections of the respiratory tract are common global causes of morbidity and mortality. Our study attempts to elucidate a novel remedy for respiratory ailments, in addition to identifying and quantifying the metabolites of *Saussurea costus* root extract (SCRE) using HPLC. Then, in vitro antiviral and in vivo lung protective effects were elucidated. The in vitro antiviral potential of SCRE was analyzed via plaque assay against the low pathogenic human coronavirus (HCoV-229E) and human influenza virus (H1N1). The value of the half maximal inhibitory concentrations (IC_50_) of SCRE against HCoV-229E and H1N1 influenza virus were 23.21 ± 1.1 and 47.6 ± 2.3 µg/mL, respectively. SCRE showed a histological improvement, namely a decrease in inducible nitric oxide synthase (iNOS) and caspase-3 immunoexpression in in vivo cyclophosphamide (CP)-induced acute lung injury (ALI). Moreover, there was a considerable decline in microRNA-let-7a gene expression and a significant rise in heme oxygenase-1 (HO-1) gene expression, with a marked decrease in the malondialdehyde (MDA) level. Molecular docking studies revealed that the major constituents of SCRE have a good affinity for caspase-3, HO-1, and iNOS proteins. In conclusion, a traditional plant SCRE could be a promising source of novel therapeutic agents for treating and protecting respiratory tract diseases. More future investigations should be carried out to reveal its efficacy clinically.

## 1. Introduction

In recent times, infections of the respiratory tract have caused a high rate of morbidity and mortality all over the world [1]. Significant attention has been drawn to such infections in the coronavirus disease 2019 (COVID-19) era. This is attributed to their extreme effects on public health, as well as on the economy [2,3]. It is well known that many viruses can cause respiratory tract infections which can cause epidemic and, in a few cases, pandemic diseases [4]. These viruses include the influenza virus and the severe acute respiratory syndrome coronavirus 2 (SARS-CoV-2) [4,5].

The development of viral strains resistant to currently available drugs is a major problem facing the control of viral infections. Many viruses are now drug-resistant due to their acquisition of resistance mutations [6]. Moreover, the inappropriate utilization of anti-influenza drugs to control infections has contributed to the occurrence of resistant influenza strains. Thus, it is essential to continuously reveal novel therapeutic alternatives with powerful activity and well-established safety [7].

Various compounds can induce acute lung injury (ALI). Cyclophosphamide (CP) is one of these compounds. It is a compound broadly prescribed to treat various malignancies, such as leukemia, lymphomas, and myeloma [8]. Furthermore, CP is utilized as an immunosuppressive drug, mainly in patients with organ transplantation. Moreover, it has therapeutic uses in systemic lupus erythematosus, nephritic syndrome, and multiple sclerosis [9].

Unfortunately, CP often leads to severe adverse effects that can predispose the patient to damage to different organs such as the heart, lungs, kidneys, and liver [10]. Oxidative and endoplasmic reticulum stress are prominent players in CP-induced lung toxicity. Oxidative stress is usually prompted by the elevated release of reactive oxygen species (ROS) [11]. Such oxidative stress is followed by fibrosis, inflammation, and apoptosis [11].

MicroRNAs (miRNAs) play an essential part in the pathophysiology of various diseases such as ALI, tuberculosis, and lung fibrosis. They target and silence protein-coding genes through three prime untranslated region (3′-UTR) elements [12,13]. The let-7 family of miRNAs is the main regulator in cell proliferation and development. It has been established that they have a potent pro-inflammatory function in many ailments, such as asthma, Alzheimer’s disease, and osteochondritis [14,15].

Heme oxygenase-1 (HO-1) is critical in cellular stress and ubiquitous in living organisms [16]. HO-1 is an enzyme with a cytoprotective role, as it catalyzes the degradation of heme to ferrous iron, carbon monoxide (CO), and biliverdin. Regarding biliverdin, it is converted to bilirubin. The two agents possess antioxidant activity [17]. HO-1 induction is regulated mainly at the transcriptional level and is mediated by nuclear transcription factor E2-related factor 2 (Nrf2), which is a major regulator of antioxidant activity [18].

Caspases are proteases present in multicellular organisms. Caspase-3 is a member of this family and is a crucial mediator of apoptosis [19]. Many inflammatory stimuli could induce the expression of inducible nitric oxide synthases (iNOS) in various cell types which synthesize nitric oxide (NO), an important pro-inflammatory mediator [20].

Malondialdehyde (MDA) is a product of the peroxidation of lipids in human cells [21]. Therefore, the rise in free radicals often results in the overproduction of MDA, an oxidative stress biomarker [22].

Plant extracts and their bioactive compounds have historically had good antiviral potential with well-established safety for controlling different viral infections [23]. The *Saussurea costus* plant is frequently employed in traditional therapies. It also has numerous bioactivities, such as antimicrobial, antilithiatic, and antioxidant properties, and a potent ability to treat and prevent a wide range of illnesses, such as cancer, diabetes, and hemorrhoids [24,25].

Our study was designed to determine and quantify the components of active *Saussurea costus* (Falc.) Lipsch. root extract (SCRE) using high-performance liquid chromatography (HPLC) and in vitro antiviral potential against the low pathogenic human coronavirus and human influenza virus H1N1. Furthermore, we designed this study to elucidate the in vivo and in silico protective effects of SCRE toward CP-induced lung injury.

## 2. Results

### 2.1. HPLC Analysis

**Figure 1:**
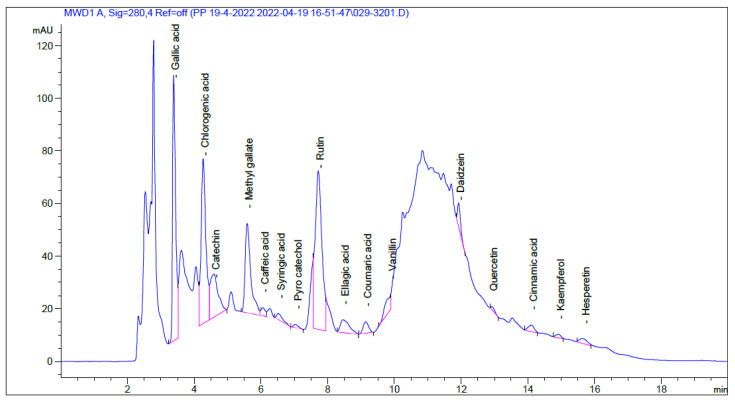
Chromatogram for the identified molecules of SCRE.

The chemical composition of SCRE was identified and quantified by utilizing HPLC. Figure 1 shows the HPLC chromatogram for the specified components in the SCRE. Ultimately, 16 components were confirmed by reference standards with a confidence level of 95%. These HPLC-identified compounds are listed in Table 1. The most abundant phenolic compounds were chlorogenic acid (90.85 μg/mL), gallic acid (51.56 μg/mL), ellagic acid (32.27 μg/mL), and methyl gallate (25.92 μg/mL). The major flavonoids were catechin (74.60 μg/mL) and rutin (61.23 μg/mL).

### 2.2. Antiviral Activity

#### 2.2.1. Cytotoxicity of SCRE on Vero-E6 Cells

**Figure 2:**
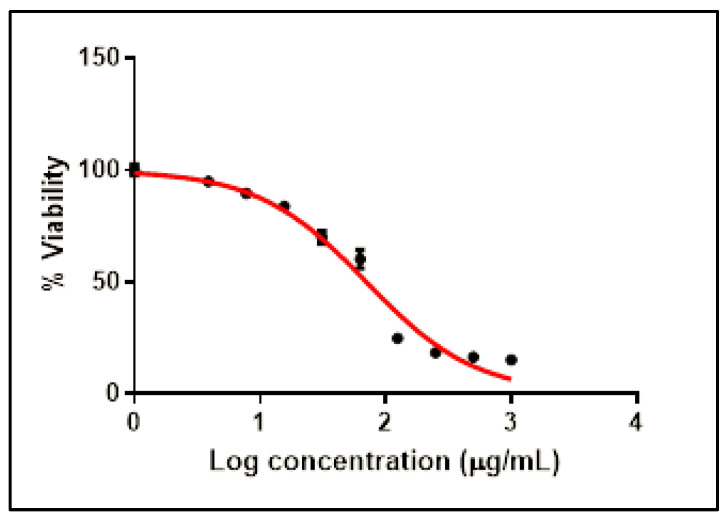
Cytotoxicity curve of SCRE (with CC_50_ of 70.36 ± 0.8 µg/mL). The results are exhibited as mean ± SD.

The CC_50_ value of SCRE was detected via MTT assay, as revealed in Figure 2.

#### 2.2.2. Antiviral Activity of SCRE

**Figure 3:**
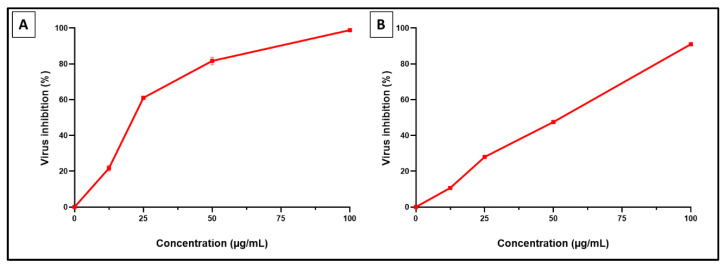
Antiviral activity of *Saussurea costus* roots on (**A**) low pathogenic human coronavirus (HCoV-229E) (with IC _50_ of 23.21 ± 1.1 µg/mL) and (**B**) human influenza virus H1N1 (with IC_50_ of 47.6 ± 2.3 µg/mL). Results are shown as mean ± SD.

The antiviral activity of SCRE against the low pathogenic coronavirus (HCoV-229E) and influenza (H1N1) virus is shown in Figure 3.

### 2.3. In Vivo Lung Protection

#### 2.3.1. Microscopical Results

**Figure 4:**
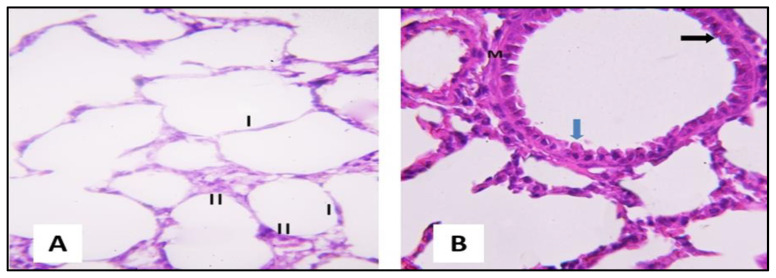
Section of a rat lung of the control (group I) showing the following features. (**A**) A normal lung architecture with thin interalveolar septa and patent alveolar sacs. A thin type I pneumocyte with flat nuclei (I) was noticed, and a small number of cuboidal type II pneumocytes with their large, rounded nuclei and vacuolated cytoplasm (II) were present at the angles of the interalveolar septa (H&E, ×400). (**B**) The bronchiole is lined by simple columnar epithelium (black arrow) and is surrounded by concentric layers of smooth muscle fibers (M). Club cell is observed in the lining of the bronchiole (blue arrow) (H&E, ×400).

H&E-stained lungs of group I presented normal lung structure, with thin interalveolar septa and clear patent alveolar cavities (Figure 4A). The alveoli were lined mostly by flat type I pneumocytes showing single flattened nuclei and a small number of cuboidal type II pneumocytes with single rounded nuclei and vacuolated cytoplasms. Alveoli were separated by thin interalveolar septa (Figure 4A). The bronchioles were lined with a simple columnar epithelium with dome-shaped club cells (Figure 4B).

**Figure 5:**
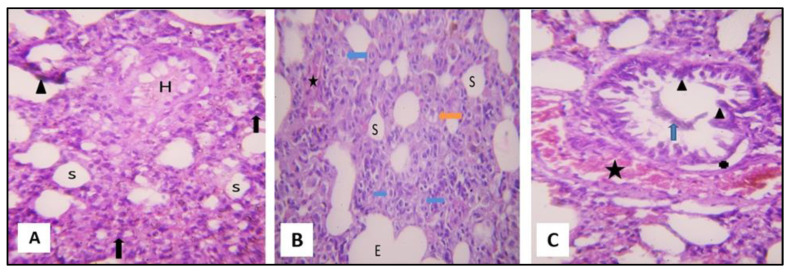
Section of a rat lung of (CP-treated) group II presenting the following features. (**A**) Thickened interalveolar septa with heavy mononuclear cellular infiltration (↑) and marked narrowing of the alveolar spaces (S). Alveolar epithelium is hardly distinguished. One regenerated alveolus lined by rounded type II pneumocytes and filled with homogenous exudate can be distinguished (H). The presence of hemosiderin-laden macrophages (▲) is noticed (H&E, ×400). (**B**) Thickened interalveolar septa with hyperplastic pneumocyte II (blue arrow), foamy macrophage (orange arrow), marked narrowing of the alveolar spaces (S), and over-dilatation of others (E). Congested blood vessel (⁕) is noticed (H&E, ×400). (**C**) The bronchiole with epithelial cells has strongly stained nuclei (▲). The bronchiolar lumen is full of exfoliated epithelial cells (blue arrow) and is marked by detachment of bronchiolar epithelium from underlying lamina propria. Congested blood vessels (⁕) are noticed with extravasated blood cells in the lung interstitium (H&E, ×400).

H&E-stained lung section of group II revealed that most lung tissues were negatively affected. The intralveolar septa were thickened; they were also populated with mononuclear cellular infiltration. Narrowed alveolar spaces were seen in some areas, whereas dilated alveoli were noticed in other areas. Extravasation of red blood cells was observed, together with homogenous acidophilic material, in the alveolar lumen. Congestion of the pulmonary vessels and hyperplastic pneumocyte type II were also noticed (Figure 5A,B). The bronchial passages showed desquamation and shedding of their lining epithelium. Desquamated cells and cellular debris were seen in the lumina of some bronchial passages. The epithelial cells that bordered the bronchiole showed strongly pigmented nuclei (Figure 5C).

**Figure 6:**
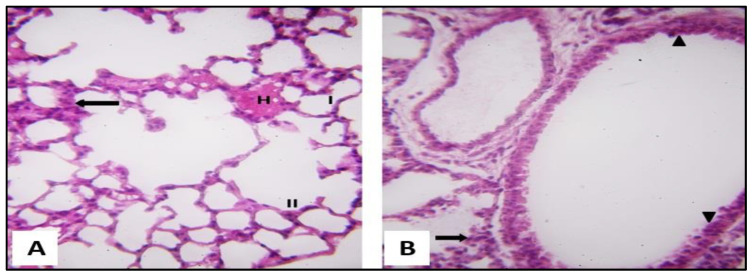
A rat lung section of group III (SCRE-treated) presenting the following features. (**A**) Thin interalveolar septa with patent alveoli. Pneumocyte type I (I) and type II (II) are noticed. Some alveoli contained homogeneous exudate (H). Few mononuclear cellular infiltrations (↑) are noticeable in some interalveolar septa (H&E, ×400). (**B**) A bronchiole shown with relatively intact epithelial lining (▲). Mononuclear cellular infiltrations (black arrow) are still present with interalveolar septa (H&E, ×400).

H&E-stained lung sections of (SCRE-treated) group III demonstrated that they were remarkably similar to those of the control, with thin interalveolar septa and clear alveolar cavities. However, some interalveolar septa still contained mononuclear cellular infiltrations. The bronchiolar epithelium appeared relatively intact (Figure 6A,B).

**Figure 7:**
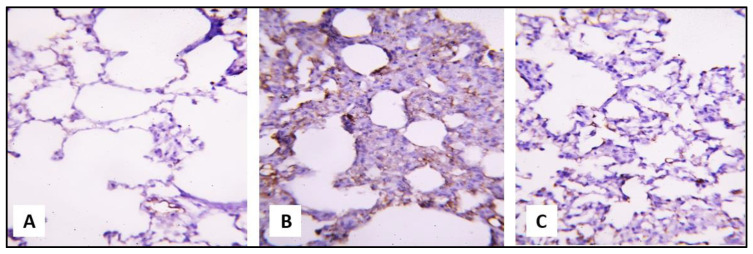
A section of a rat lung showing the following features. (**A**) A weakly positive brown cytoplasm reaction in the alveolar epithelial lining of the control (iNOS immunostaining, ×400). (**B**) A dense, positively brown cytoplasm reaction in the alveolar epithelial lining, inflammatory interstitial cells, and endothelial cells of group II (iNOS immunostaining, ×400). (**C**) A weakly positive brown cytoplasm reaction in the alveolar epithelial lining and endothelial cells of group III (iNOS immunostaining, ×400).

**Figure 8:**
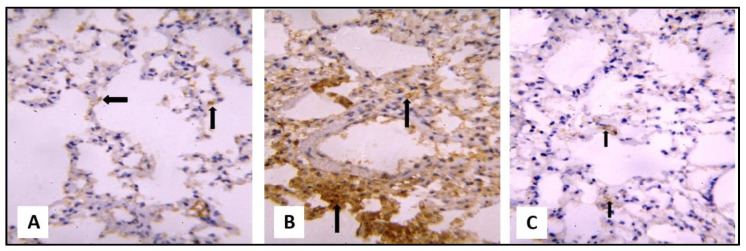
A section of rat lung showing the following features. (**A**) A weakly positive brown cytoplasm reaction along the alveolar epithelial lining of the control group (caspase-3 immunostaining, ×400). (**B**) A dense, positive brown cytoplasmic reaction in the exfoliated cells and interstitial inflammatory cells (black arrow) of group II (caspase-3 immunostaining, ×400). (**C**) A weakly positive brown cytoplasm reaction in the alveolar epithelial lining of group III (caspase-3 immunostaining, ×400).

Examination of the iNOS- and caspase-3 immune-stained cells showed a weakly positive brown cytoplasm reaction in alveolar epithelial lining of the control and group III. On the other hand, a dense positive brown cytoplasmic reaction was evident in group II (Figure 7 and Figure 8).

**Figure 9:**
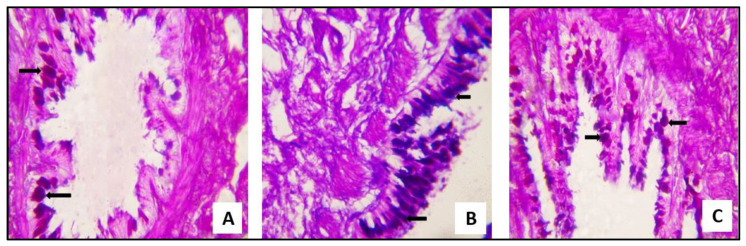
A section of rat lung showing the following features. (**A**) PAS-positive cells (↑) in the epithelium of a large bronchus of the control group (PAS stain, ×400). (**B**) An apparent increase in the PAS-positive cells in the bronchial passage lining epithelium of group II (PAS stain, ×400). (**C**) The PAS-positive cells in the lining epithelium of a large bronchial passage of group III is relatively comparable to the control group (PAS stain, ×400).

The apparent rise in PAS-positive cells in the large bronchial passage lining relative to the control was noticed in group II PAS-stained sections (Figure 9).

#### 2.3.2. Biochemical Results

##### SCRE Reduced Lipid Peroxidation and Oxidative Stress in CP-Treated Rats

**Figure 10:**
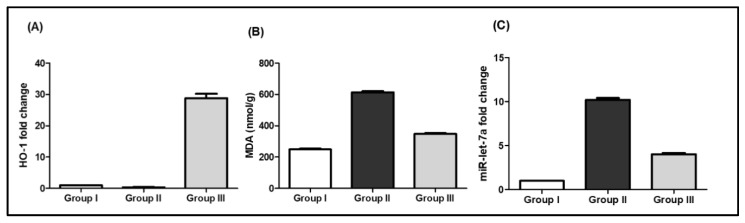
Levels of the studied markers in the lung tissues of the different groups (**A**) Relative expression of HO-1 mRNA. (**B**) MDA levels. (**C**) Relative expression of miR-let-7a. Data are expressed as mean ± SD.

To assess the effects of CP on lipid peroxidation and oxidative stress in the lung of rats, in addition to the protective effects of SCRE, the HO-1 expression and the concentration of MDA were determined. CP administration decreased the HO-1 expression (Figure 10A) and dramatically raised the concentration of MDA (Figure 10B) in the lung of rats relative to the control (*p* < 0.05). The administration of SCRE significantly upregulated HO-1 mRNA and decreased the MDA in the CP-treated rat lung (*p* < 0.05).

##### miR-let-7a Expression in Response to CP and SCRE

CP-induced lung injury and inflammatory state showed a significant upregulation of miR-let-7a relative to the control group. Following the SCRE administration, miR-let-7a expression significantly decreased (*p* < 0.05, Figure 10C).

### 2.4. Molecular Docking Studies

**Figure 11:**
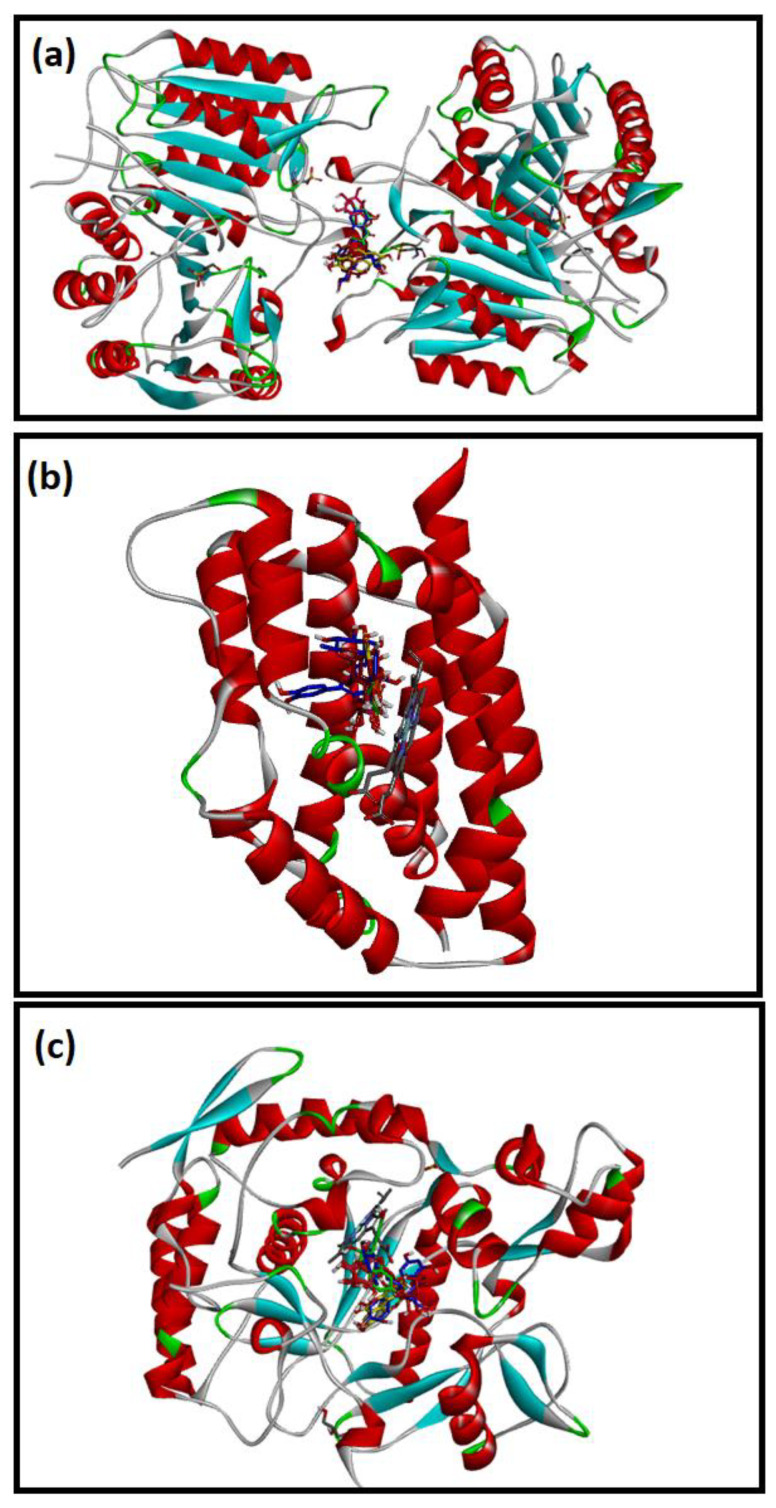
Overlay of docked rutin (blue), catechin (green), chlorogenic acid (red), ellagic acid (yellow), methyl gallate (pink), and gallic acid (orange) into (**a**) the caspase-3 protein (Code: 3DEI), (**b**) HO-1 (Code: 3CZY), and (**c**) iNOS (Code: 1NSI).

The molecular mechanisms for the main constituents of SCRE, namely, RUT, GAL, CHL, GAT, CAT, and ELL on caspase-3, HO-1, and iNOS proteins, were studied by employing a molecular docking strategy. The aforementioned ligands revealed strong affinity binding to the target of interest, reflected by the obtained docking scores (Table 2). All ligands accommodated the same binding site in the three proteins (Figure 11).

**Figure 12:**
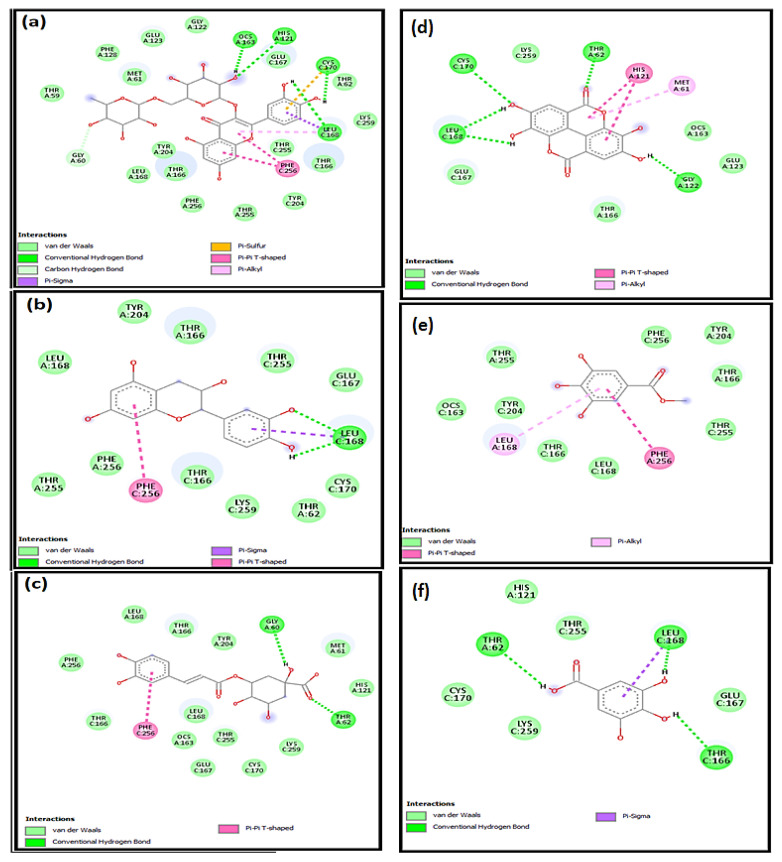
The 2D schematic interactions of docked (**a**) RUT, (**b**) CAT, (**c**) CHL, (**d**) ELL, (**e**) GAT, and (**f**) GAL into the caspase-3 protein (Code: 3DEI).

Docking inside the caspase-3 protein showed four H-bonds with RUT, whereas CAT and CHL formed only two. Similarly, ELL showed four H-bonds, whereas GAL formed three H-bonds, and GAT showed no H-bonds with caspase-3 (Figure 12). The **π–π** interaction with Phe256 was observed with the aromatic system of RUT, CAT, CHL, and GAT, whereas the aromatic system in ELL caused the π–π interaction with His121. The affinity to caspase-3 was reflected in terms of binding scores in Table 2.

**Figure 13:**
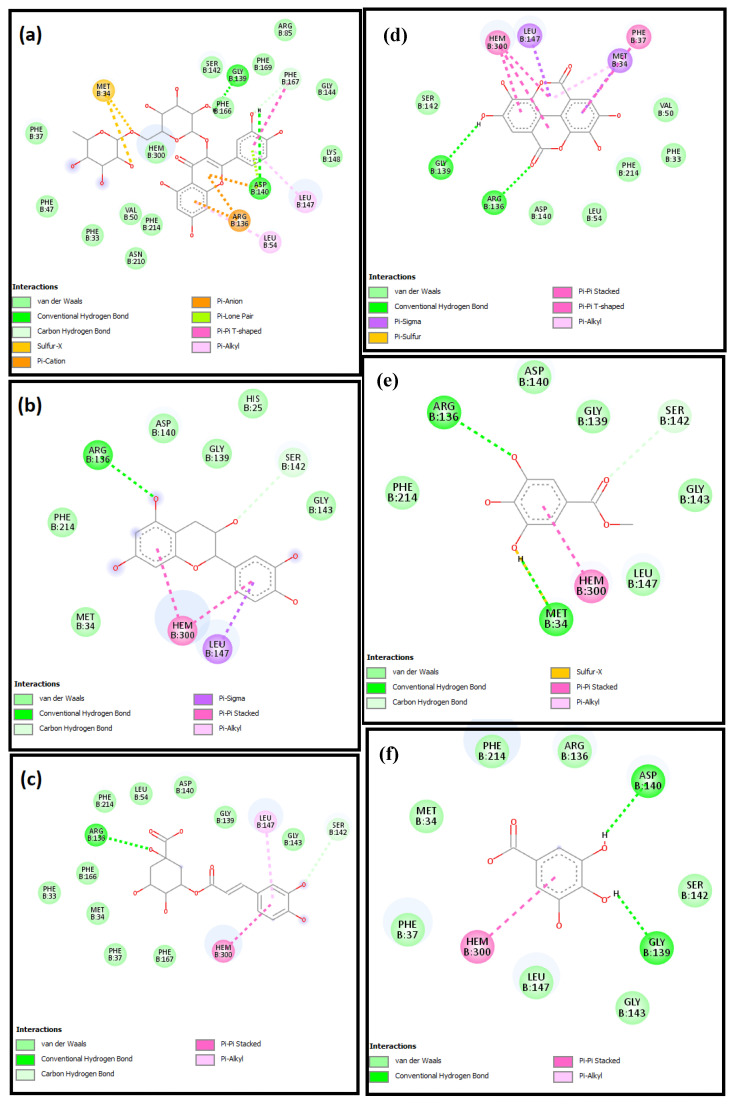
The 2D schematic interactions of docked (**a**) RUT, (**b**) CAT, (**c**) CHL, (**d**) ELL, (**e**) GAT, and (**f**) CAT with human HO-1 (Code: 3CZY).

Inside the active site of human HO-1, the six major components of the active extract were inserted near the heme molecule (Figure 11). RUT showed different types of interactions, including two H-bonds, π–anion, π–cation, and π–π interaction, whereas CAT and CHL showed H-bonds and π–π interaction (Figure 13). Similarly, ELL showed H-bonds, π-sulfur, and π–π interaction, whereas GAL and CAT showed H-bonds and **π–π** interaction only. All ligands showed certain interaction with heme molecule, and the net interaction score is presented in Table 2.

**Figure 14:**
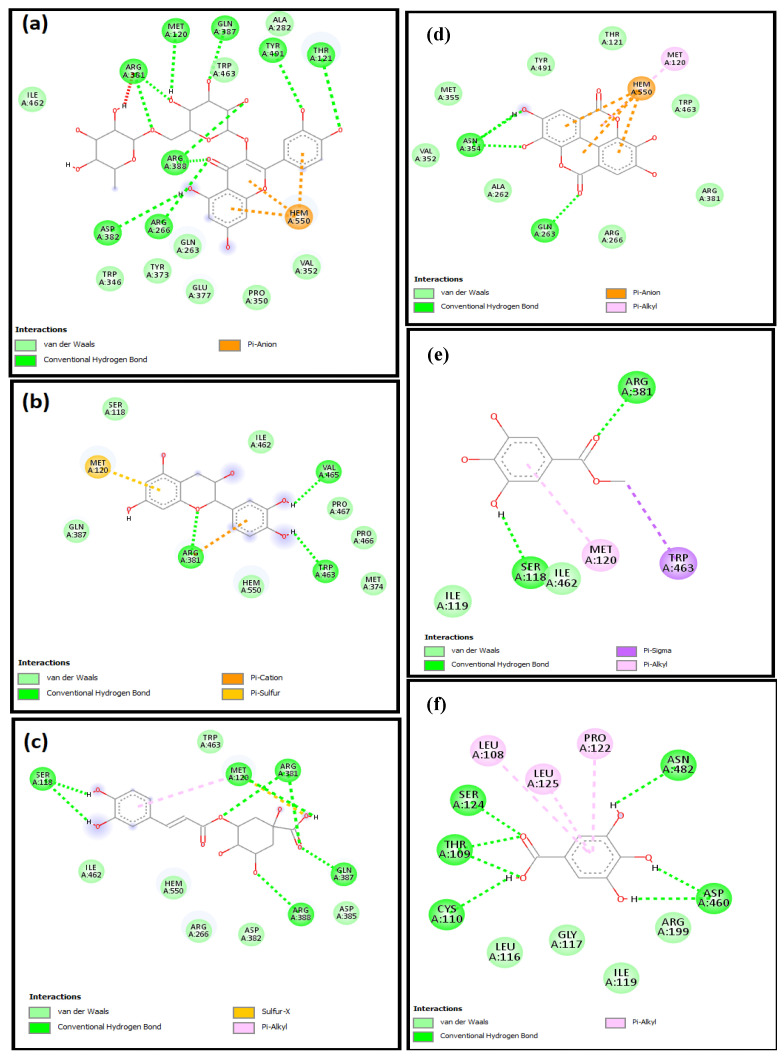
The 2D schematic interactions of docked (**a**) RUT, (**b**) CAT, (**c**) CHL, (**d**) ELL, (**e**) GAT, and (**f**) CAT with human inducible nitric oxide synthase (Code: 1NSI).

Furthermore, docking in the iNOS was performed, and all compounds were positioned near the heme molecule (Figure 11). RUT and ELL showed direct interactions with the heme molecule, and the H-bonds showed direct interactions with the nearby amino acids (Figure 14). On the other hand, CAT, CHL, GAT, and CAT showed no direct interaction with the heme and depended mainly on the H-bond interactions.

**Figure 15:**
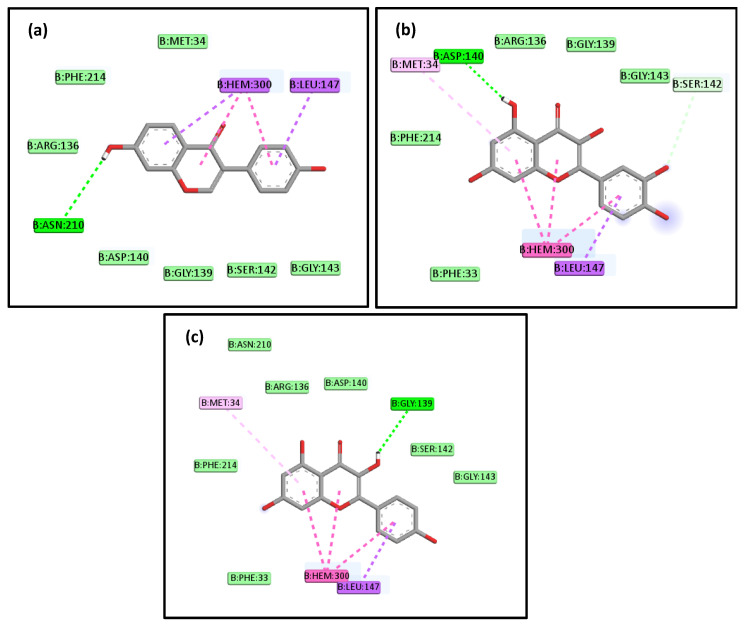
The 2D schematic interactions of docked daidzein (**a**), quercetin (**b**), and kaempferol (**c**) with human HO-1 (Code: 3CZY).

In addition, other components of the active extract that were found in low concentrations also demonstrated good affinity to the three studied targets. Interestingly, among these constituents, daidzein, quercetin, and kaempferol showed higher affinity to HO-1 with binding affinities of −9.0, −9.0, and −8.9 kcal/mol, respectively (Figure 15). This affinity is even greater than those shown by the most abundant compounds, which may suggest their contribution to the overall activity of the total extract.

The components of the active *Saussurea costus* root extract SCRE showed a good affinity for the caspase-3, HO-1, and iNOS proteins, as previously shown in the in vivo experiment.

## 3. Discussion

The in vitro antiviral potential of SCRE was explored against the low pathogenic coronavirus (HCoV-229E) and (H1N1) virus via plaque assay. This assay is a common method for enumerating infectious viruses [26]. Its principle is to detect the count of the produced plaques in the infected cells before and after treatment with the tested agent [27]. In the present study, SCRE revealed IC_50_ values of 23.21 ± 1.1 µg/mL and 47.6 ± 2.3 µg/mL against HCoV-229E and H1N1 influenza virus, respectively. The IC_50_ value represents the required drug concentration to produce an in vitro 50% inhibition of the tested virus [27]. Some researchers have reported the anti-influenza potential of certain plant extracts such as the *Artemisia cina* flower extract [7,28].

In the present study, the lungs of group II showed focal disruption and shedding of the epithelial lining of the bronchial passages. Such findings are consistent with Shokrzadeh et al. [29], who noticed the presence of lymphocytes, neutrophils, cell debris, and hyperplastic pneumocytes in vast distal air gaps. Moreover, congestion of the pulmonary vessels, oedema, extravasation of RBCs in the interstitium, thickened interalveolar septa with heavy mononuclear cellular infiltration, and pneumocyte II hyperplasia were noticed in rats of group II. These findings coincide with those of Bhattacharjee et al. [30], who noticed alveolar cell injuries, thickness in alveolar septa, polymorphonuclear cells, and erythrocytes in the alveolar lumen with edema in the epithelial cell structure due to CP administration.

Here, the lungs of rats that received CP (group II) showed increased expression of iNOS immunohistochemistry, which is in agreement with the findings of Abdel Latif et al. [31], who described intense, brown-colored iNOS immune reactive cells in the interalveolar septa in the CP-treated group. Speyer et al. [32] stated that there is potential to minimize the lung injury effect induced by endogenous nitric oxide (NO) through the reduction in the neutrophil recruitment by lowering sticky contacts with the endothelium and altering chemokine production.

In the present study, the lungs of group II showed an increased immune expression of caspase 3. Amirkhizi et al. [33] referred to the occurrence of cell death as ROS, which has the ability to destroy many components of the cells and decrease the antioxidant enzymes. ROS stimulate the peroxidation of lipids, which adversely affects the cell membrane integrity and predisposes the cell to cell death [33]. Previous research has shown that SCRE exhibited various pharmacological bioactivities, such as anti-inflammatory, anticancer, and antioxidant potentials [34].

Zahara et al. [35] stated that *S. costus* has anti-inflammatory activity. Lee et al. [36] found that *S. costus* inhibited cytokine-induced neutrophil chemotactic factor 30. This illustrates the marked decrease in the inflammatory cells in the SCRE-treated group in our study. Choi et al. [37] verified the impact of *S. costus* on apoptosis-related gene mRNA and protein levels. This could explain the decreased caspase-3 level in the SCRE-treated group.

It is well-established that HO-1 has antioxidant, anti-inflammatory, antiproliferative, and immunomodulatory impacts [38]. In this study, we elucidated the level of HO-1 in the rats after the administration of CP, and there was a marked decrease in its expression. The effect of CP administration was attenuated by treatment with SCRE. In agreement with Choi et al. [18], SCRE induced HO-1 mRNA expression, proving its cytoprotective role against inflammation.

As further proof of the promising antioxidant role of SCRE, the MDA level was measured as an indicator of oxidative stress. This study showed that the MDA level was enhanced in the lung of CP-administered animals, whereas rats with CP-induced ALI with SCRE demonstrated a noticeable decline in the MDA level, demonstrating the potent antioxidant activity of SCRE. This result is boosted by previous studies that proved the reduction in oxidative stress markers following the administration of SCRE [18,39].

On the other hand, previous studies have shown that miR-let-7a has a pro-inflammatory role. In our study, we evaluated the expression of miR-let-7a in CP-induced ALI in rats, and there was a significant increase in its expression, demonstrating its pro-inflammatory role. Such findings are in line with the findings of Polikepahad et al. [40]. They illustrated the pro-inflammatory role of miR-let-7a in experimental asthma. Furthermore, other consistent results were reported by Sui et al. [14], as they proved that miR-let-7a inhibitor substantially decreased protein expression amounts of multiple inflammation biomarkers such as tumor necrosis factor-alpha (TNF-α) and interleukins (1β and 6) in osteoarthritis. Moreover, the SCRE-treated group revealed a notable reduction in the miR-let-7a expression, suggesting a putative mechanism for the protective role of SCRE against inflammation in CP-induced ALI.

## 4. Materials and Methods

### 4.1. Plant Preparation and High-Performance Liquid Chromatography (HPLC) Analysis

*Saussurea costus* (Falc.) Lipsch. root was obtained from the local market “El-Gelany” of Menoufia in January 2022. Dr Esraa Ammar from the Plant Ecology Department, Faculty of Science, Tanta University, kindly verified the plant identification. A voucher specimen (PG-A-SC-W-12) was kept at the Plant Ecology Department. *Saussurea costus* root powder (300 g) was extracted with 70% methanol in water three times. Next, the aqueous extract was evaporated under reduced pressure to yield SCRE residue (23.09 g).

HPLC analysis of the SCRE was performed according to Seliem et al. [41] with some modifications. The SCRE was analyzed using an Agilent 1260 series instrument and Eclipse C18 column (4.6 mm × 250 mm i.d., 5 µm). Separation was performed at a flow rate of 0.9 mL/min. The mobile phase consisted of water (reservoir A) and 0.5% trifluoroacetic acid in acetonitrile (reservoir B) at a concentration of 0.1%. The mobile phase was sequentially programmed with a linear gradient as follows: 0 min (82%A); 0–5 min (80%A); 5–8 min (60%A); 8–12 min (60%A); 12–15 min (82%A); 15–16 min (82%A); and 16–20 (82%A). A multi-wavelength UV detector was used for detection at 280 nm. The injection volume for each sample solution was 5 μL. The column temperature was retained at 40 °C.

### 4.2. Chemicals and Solvents

All chemicals, reagents, and solvents were obtained from Merck, Rahway, NJ, USA.

### 4.3. In Vitro Antiviral Activity

#### 4.3.1. Viruses and Cells

Vero-E6 cells (Vacsera, Egypt) are employed for the propagation of the low pathogenic human coronavirus (HCoV-229E) and influenza virus (A/Egypt/099/2020) (H1N1). The medium was Dulbecco’s modified Eagle’s medium (DMEM) with 10% fetal bovine serum (FBS) (Merck, London, UK) and 1% penicillin/streptomycin mixture (Merck, London, UK). The incubation conditions were 37 °C in the presence of 5% CO_2_. In order to produce viral stocks, the cells were inoculated into an infection medium consisting of DMEM, 2% FBS, 1% penicillin/streptomycin, and 1% trypsin (treated with L-1-tosylamido-2-phenylethyl chloromethyl ketone). The viruses and the cells were incubated for two hours. Then, the medium was substituted with a new one and incubated for three days. The supernatant was then centrifuged to discard the debris of the cells. In the end, it was titrated via plaque assay [42].

#### 4.3.2. MTT Cytotoxicity Test

This assay was performed to detect the half-maximal cytotoxic concentration (CC_50_) of SCRE, as described before [42]. Using an ELISA reader (Sunrise Tecan, Switzerland), we measured the absorbance (A) of produced formazan at an optical density (OD) of 540 nm. The % cytotoxicity was determined as in the formula:

$$\%\;\mathrm{cytotoxicity}=\frac{\;A\;\mathrm{of}\;\mathrm{cells}\;\mathrm{without}\;\mathrm{treatment}-A\;\mathrm{of}\;\mathrm{treated}\;\mathrm{cells}}{A\;\mathrm{of}\;\mathrm{cells}\;\mathrm{without}\;\mathrm{treatment}\;}\times 100$$

where A is the absorbance.

Then, we constructed a curve between the cytotoxicity percentage and the concentration.

#### 4.3.3. Plaque Assay

This test was performed as reported previously [7,42] using Vero-E6 cells to reveal the antiviral potential of SCRE. In brief, the diluted viruses (in DMEM) were added to Vero-E6 cells and incubated for one hour at 37 °C. Then, the supernatant was discarded, and the cells were covered with DMEM with 2% agarose containing SCRE. After solidification of the agarose, the six-well plates were incubated at 37 °C in 5% CO_2_ for three days. Then, 10% formalin was added to the wells for one hour for fixation and removed by washing the wells with water. Finally, 0.1% crystal violet solution was added for staining, splashed with water, and allowed to dry. The formed plaques were non-stained spots against a violet background. Each plate had a control well of untreated, infected Vero-E6 cells. The % inhibition of the production of plaques was determined by counting the formed plaques according to the following formula:

$$\%\;\mathrm{inhibition}=\frac{\mathrm{Untreated}\;\mathrm{viral}\;\mathrm{count}-\mathrm{treated}\;\mathrm{viral}\;\mathrm{count}}{\mathrm{untreated}\;\mathrm{viral}\;\mathrm{count}}\times 100$$

### 4.4. In Vivo SCRE Lung Protection

#### 4.4.1. Animals

Forty-five male Wister rats (180–210 g) were obtained from Ain shams. Rats were preserved in controlled temperature, humidity, and 12 h light–dark cycles. They were provided filtered water and an ad libitum diet. All experiments were conducted in line with the guidelines of the international council of Harmonization (ICH) under Federal-Wide Assurance No. FWA 000017585. The Ain Shams Faculty of Medicine Ethical Committee approved the study with the following authorization number: “FMASU R209/2022”.

#### 4.4.2. Experimental Protocol

Rats were randomly classified into three groups (n = 15):

- Group I (control) was given 0.5 % carboxymethyl cellulose (CMC) orally for 10 days and only one intraperitoneal (IP) saline injection on the seventh day.
- Group II (CP-treated) was given 0.5 % CMC for 10 days and a single IP CP injection (200 mg/kg) on the seventh day [29].
- Group III (SCRE-treated) was given 100 mg/kg SCRE in 0.5 % CMC for 10 days and a single IP CP injection (200 mg/kg) on the seventh day [43,44].

In the end, all rats were anesthetized and euthanized by cervical dislocation. Afterwards, the lungs were obtained and washed with saline. The right lung was homogenized to be analyzed biochemically. The left lungs were placed in 10% formalin for 72 h and subjected to histological and immunohistochemical studies [45].

#### 4.4.3. Histological and Immunohistochemical Studies

Histological examination was performed by staining with hematoxylin and eosin (H&E) and periodic acid–Schiff (PAS) stains, as previously described [45,46]. Regarding the immunohistochemical staining, it was carried out using a primary anti-caspase-3 antibody (Abcam, Cambridge, UK), as described before, via the avidin–biotin complex (ABC) technique [46]. As previously reported, the immunohistochemical staining of iNOS was performed using rat monoclonal antibody (1:500 dilution, Transduction Laboratories, CA, USA) [47]. Negative controls were provided by skipping the incubation with the primary antibody. Positive immunoreactivity (brown staining) for caspase and iNOS staining was visualized microscopically.

#### 4.4.4. Biochemical Studies

##### Detection of MDA Level

Lipid peroxidation was assayed in the lungs by a specific MDA colorimetric assay kit, catalogue no.: E-BC-K025-S (Elabscience Biotechnology, USA).

##### Detection of the Relative Gene Expression of miR-Let7a and HO-1

As per the manufacturer’s protocol, total mRNA and miRNAs were extracted with a miReasy Mini Kit (Qiagen, Hilden, Germany). The complementary DNA (cDNA) was prepared via a reverse transcription reaction from miScript RT Kit (Qiagen, Hilden, Germany). The miRNA and mRNA genes were amplified via miScript Syber green master mix and QuantiTect SYBR Green PCR Kit, respectively (Qiagen, Hilden, Germany). The 5 plex Rotor-Gene PCR Analyzer (Qiagen, Hilden, Germany).

The relative expression level (fold change) for the miR-let7a and HO-1 gene was normalized to SNORA11 and β-actin internal controls, respectively, and relative to the calibrator (negative control sample), they were calculated using the equation 2^−∆∆Ct^ test [48,49]. The relative gene expression of miR-let7a and HO-1 was determined using the miScript primer assay (miR-let-7a), catalogue number: MS00031220, and QuantiTect primer assay (HO-1), catalogue number: 249900 (Qiagen, Germany). Regarding the housekeeping genes, SNORA11, miScript primer assay with catalogue number 218300 and β-actin gene, ACTB_ 1_SG QuantiTect primer assay with catalogue number 249900, were used (Qiagen, Hilden, Germany).

### 4.5. In Silico Lung Protective Potential of SCRE

**Figure 16:**
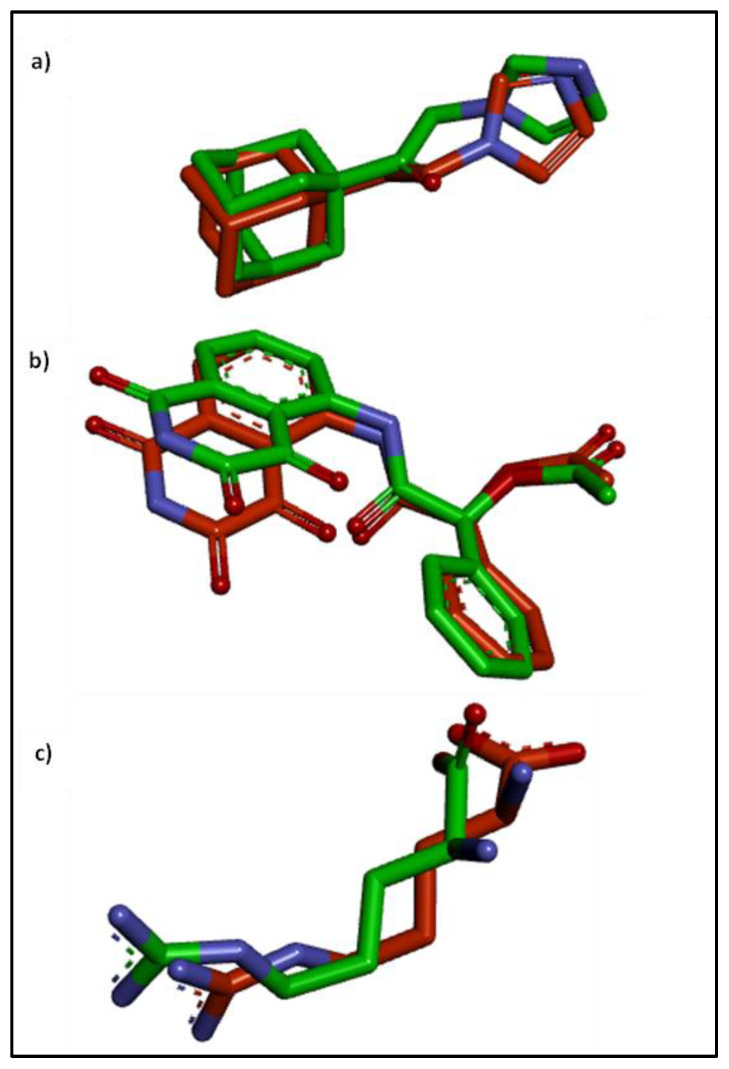
Overlay of the docked and co-crystalized ligand of (**a**) HO-1 (RMSD = 1 Å), (**b**) caspase-3 (RMSD = 1.118 Å), and (**c**) inducible nitric oxide synthase (RMSD = 1.496 Å) enzymes.

The crystal structure of caspase-3 (Code:3DEI) [50], human HO-1 (Code: 3CZY) [51], and human iNOS (Code: 1NSI) [52] were retrieved from the protein data bank. The docking study was carried out on rutin (RUT), gallic acid (GAL), chlorogenic acid (CHL), methyl gallate (GAT), catechin (CAT), and ellagic acid (ELL) using AutoDock Vina [53]. Ligand structures were drawn into Marvin Sketch V22.2 [54] and were exported in their most energetically favored conformers. The docking simulation was conducted following our preceding study steps. The center and size of the grid box to define the active site for each receptor are listed in Table 2. The 3D visualization and 2D schematic presentation were produced by the Discovery Studio client [55]. The docking simulation was conducted following our preceding study steps after being validated by redocking the co-crystalized ligands (Figure 16).

### 4.6. Statistics

The obtained data are exhibited as mean ± standard deviation (SD). The significance of the difference among the groups was analyzed using ANOVA followed by a post hoc test (Tukey). The difference was supposed to be significant if *p* < 0.05 by Prism (GraphPad Software, Inc., San Diego, CA, USA).

## 5. Conclusions

In this study, the antiviral activity of *Saussurea costus* root was revealed against the low pathogenic human coronavirus (HCoV-229E) and influenza virus (H1N1) with IC_50_ values of 23.21 ± 1.1 µg/mL and 47.6 ± 2.3 µg/mL, respectively. Furthermore, SCRE provided potential protection against CP-induced ALI, as revealed by the histological, immunohistochemical, biochemical, and in silico investigations. The current study highlights the potential applicability of the traditional *Saussurea costus* root in treating respiratory diseases.

## Figures and Tables

**Table 1:** Analysis of the phenolic and flavonoid components of SCRE using HPLC.

Identified Compound	RT (Min)	Area (mAU*s)	Conc (µg/mL)	Conc (µg/g)
Gallic acid	3.385	636.15	51.56	865.05
Chlorogenic acid	4.269	625.20	90.85	1524.30
Catechin	4.596	268.25	74.60	1251.74
Methyl gallate	5.589	397.33	25.92	434.83
Caffeic acid	6.055	27.85	2.14	35.84
Syringic acid	6.522	32.05	2.63	44.10
Pyro catechol	7.028	13.91	1.98	33.15
Rutin	7.722	832.21	61.23	1027.28
Ellagic acid	8.464	92.95	32.27	541.44
Coumaric acid	9.144	49.85	1.64	27.44
Vanillin	9.891	70.48	2.69	45.20
Ferulic acid	10.237	0.00	0.00	0.00
Naringenin	10.490	0.00	0.00	0.00
Daidzein	11.927	54.06	3.74	62.69
Quercetin	12.925	10.20	1.25	21.02
Cinnamic acid	14.111	30.51	0.65	10.86
Apigenin	14.496	0.00	0.00	0.00
Kaempferol	14.924	14.03	1.49	25.04
Hesperetin	15.684	30.45	1.78	29.93

**Table 2:** The grid box parameter (center and size) and docking binding affinity (Kcal/mL) of RUT, GAL, CHL, ELL, GAT, and CAT for caspase-3, HO-1, and iNOS proteins.

Receptor	Grid Box (x, y, z)		Affinity (kcal/mol)					
	Center	Size	RUT	GAL	CHL	ELL	GAT	CAT
Caspase 3	−46.6, 15.4, −22.4	19.7, 15.6, 18.1	−8.9	−5.0	−7.4	−7.5	−5.0	−7.8
HO-1	27.0, 16.2, −37.6	21.7, 21.4, 20.0	−7.5	−5.9	−8.0	−7.0	−6.0	−8.5
iNOS	12.5, 59.9, 20.9	30.5, 22.9, 23.5	−10.6	−6.9	−8.0	−7.1	−6.7	−7.6

## Data Availability

The authors confirm that the data supporting this study are available within the article.
